# The impact of gravity on perceived object height

**DOI:** 10.1038/s41526-024-00430-3

**Published:** 2024-10-04

**Authors:** Björn Jörges, Nils Bury, Meaghan McManus, Ambika Bansal, Robert S. Allison, Michael Jenkin, Laurence R. Harris

**Affiliations:** 1 Center for Vision Research, York University, Toronto, ON Canada; 2 Institute of Visual Computing, Hochschule Bonn-Rhein-Sieg, St. Augustin, Germany; 3 Department of Experimental Psychology, Justus Liebig University Giessen, Giessen, Germany

**Keywords:** Human behaviour, Psychology

## Abstract

Altering posture relative to the direction of gravity, or exposure to microgravity has been shown to affect many aspects of perception, including size perception. Our aims in this study were to investigate whether changes in posture and long-term exposure to microgravity bias the visual perception of object height and to test whether any such biases are accompanied by changes in precision. We also explored the possibility of sex/gender differences. Two cohorts of participants (12 astronauts and 20 controls, 50% women) varied the size of a virtual square in a simulated corridor until it was perceived to match a reference stick held in their hands. Astronauts performed the task before, twice during, and twice after an extended stay onboard the International Space Station. On Earth, they performed the task of sitting upright and lying supine. Earth-bound controls also completed the task five times with test sessions spaced similarly to the astronauts; to simulate the microgravity sessions on the ISS they lay supine. In contrast to earlier studies, we found no immediate effect of microgravity exposure on perceived object height. However, astronauts robustly underestimated the height of the square relative to the haptic reference and these estimates were significantly smaller 60 days or more after their return to Earth. No differences were found in the precision of the astronauts’ judgments. Controls underestimated the height of the square when supine relative to sitting in their first test session (simulating Pre-Flight) but not in later sessions. While these results are largely inconsistent with previous results in the literature, a posture-dependent effect of simulated eye height might provide a unifying explanation. We were unable to make any firm statements related to sex/gender differences. We conclude that no countermeasures are required to mitigate the acute effects of microgravity exposure on object height perception. However, space travelers should be warned about late-emerging and potentially long-lasting changes in this perceptual skill.

## Introduction

The height and width of objects around us provide cues about the scale of our environment. This helps us complete tasks such as estimating distances, reaching for and grasping objects, and deciding if our car will fit in a parking space. However, our perception of the size and width of an object is dependent on the distance at which it is perceived^1–4^. Gravity affects size perception. Studies conducted both in the microgravity phase of parabolic flight as well as in the microgravity conditions on the International Space Station (ISS)^5–9^ showed that size and distance estimates are affected by microgravity. On the ISS, astronauts set the height of a cube as too short when matching its height to its width^6^ suggesting that its height was seen as elongated. Additionally, the distance from the front to the back of a cube was underestimated in microgravity^5,6^ relative to judgments in Earth-normal gravity, suggesting compression of perceptual space. Another study investigating how wide an aperture had to be for the participant to feel that they could fit through^10^, found that the minimal width was narrower in the microgravity phase of parabolic flight than under Earth-normal gravity conditions. They concluded that participants either perceived themselves to be smaller or the aperture to be wider in short-duration microgravity. Crucially, they found that the subjective level of the eyes was also lower in short-duration microgravity, which may play a role in explaining their findings. Note, however, that this effect is not always found. Another study using the same paradigm^11^ comparing the critical aperture during long-term space flight onboard the ISS to performance on Earth found no significant difference, possibly due to a smaller sample size or to adaptation to microgravity conditions. However, direct measurements of the perceived size of objects some distance away from the observer are lacking.

The role of gravity in the perception of size and distance can be studied on Earth by manipulating the relation between gravity and the body by comparing judgments made while in different body postures. Participants in Harris and Mander’s study^12^ compared the perceived length of a reference stick held aligned with the long axis of their body with a vertical line projected on the wall of a visually textured room that could be pitched independently of the observer’s orientation. When the participant was strapped into a chair tilted to a supine position while the relation between the body and the visual stimulus was maintained, the projected line needed to be made 9% longer than when seated upright. Interestingly, approximately the same distortion was observed when participants were physically upright but the room was pitched by 90° to evoke only the perception of being supine^12^. Similar results were also reported by Kim, McManus, and Harris^13^ using a virtual reality display that provided disparity cues and a physical reference stick, where participants needed to make the target 5.4% larger when supine and 10.1% larger when prone compared to when upright. That is, in both these studies, as in the microgravity studies, visual objects were seen as smaller when supine (while keeping the relation between the body and the visual stimulus the same) compared to when upright and had to be made larger to match the length of a physical reference. Higashiyama and Adachi^14^ inverted the relationship between the body and gravity by having participants bend forward and view physical targets through their legs; responses were given as verbal reports of the perceived distance. Perceived size was increasingly underestimated the further away the target was when participants viewed the target through their legs. However, this manipulation significantly affected participant eye height and proprioception as well as inverted the scene, complicating the attribution of the effect to gravity. Their further experiments with scene-inverting goggles^14^ suggested it was the postural change not the inversion of the scene that was critical. Importantly, postural manipulations do not only change the relation between gravity and the body but also change somatosensory and tactile cues – perhaps these also contribute to some of the observed changes in size perception. A recent study^15^ manipulated posture both on land and underwater at neutral buoyancy thus removing somatosensory cues to posture. This study found no differences between experimental conditions suggesting the role of somatosensory postural cues was negligible in determining perceived object size.

While there is mounting evidence that gravity is involved in the perception of size, a possible mechanism remains unclear. Clément and colleagues^16^ proposed that in microgravity there might be a rescaling of visual space. Similarly, it has been suggested that gravity might offer a reference frame in which to interpret visual input^12^. Such a reference frame would be altered when the relation between the body and gravity was manipulated or unavailable in microgravity^17^. If the vestibular system plays a direct role in size estimation, we would expect that in unusual gravity conditions, uncertainty should increase, and judgments should become less precise, especially since vestibular signals concerning the direction of gravity are noisier when lying supine than when upright^18^, in line with Barnett-Cowan et al.’s^19^ finding that precision in judging the subjective visual vertical (SVV) and the perceptual upright (PU) was worse when participants were lying right side down in comparison to when they were sitting upright. Confirming these postural effects and looking for posture- and microgravity-related changes in precision were therefore two goals of the present study.

Another objective was to look for possible sex/gender differences. The idea that there might be gender-related differences in size perception is based on some observations of differences between women and men in visual^20,21^, vestibular^22,23^, and visuo-vestibular^19^ tasks as well as the relative prevalence of vestibular disorders^24^ in women.

In order to measure the effect of gravity on perceived size during normal viewing of a remote object we estimated perceived size in the microgravity of space using a similar methodology to that used by Harris and Mander^12^ and Kim et al.^13^ Participants compared the perceived visual height of a square simulated as being a few meters away in virtual reality with the length of a physical reference stick held aligned with the long axis of their body. In line with previous results, our hypothesis was that participants would set the height of the square larger when lying supine and when in microgravity relative to their settings when upright on Earth, indicating an underestimation of the square’s perceived height. Our additional hypotheses were that the increased noise of the vestibular signal while supine^18^ or in microgravity, would decrease the precision of participants’ judgments, providing evidence for direct visual-vestibular interaction in depth perception. Our further hypotheses were that these effects would persist for a short period of time after return to Earth, with performance eventually returning to pre-flight baseline levels.

## Results

### Astronauts

#### Accuracy

The intercept for the model used to test for accuracy differences in the astronauts was 1.43 (95% CI = [1.15; 1.67]). There was no significant difference between Sitting and Supine within any of the test sessions. However, between sessions PSE ratios were significantly higher in the Sitting posture at Late Post-Flight than in the Sitting posture at Pre-Flight (by 0.37, 95% CI = [0.17;0.57]), higher in the Supine posture at Late Post-Flight than in the Sitting posture at Pre-Flight (by 0.3, 95% CI = [0.12;0.49]). When testing for an interaction between the astronaut group and the control group, we found that this difference was significant only for the Sitting posture (0.53, 95% CI = [0.01;1.02]), not for the Lying Supine posture (0.42, 95% CI = [0.03;0.93]).

**Fig. 1:**
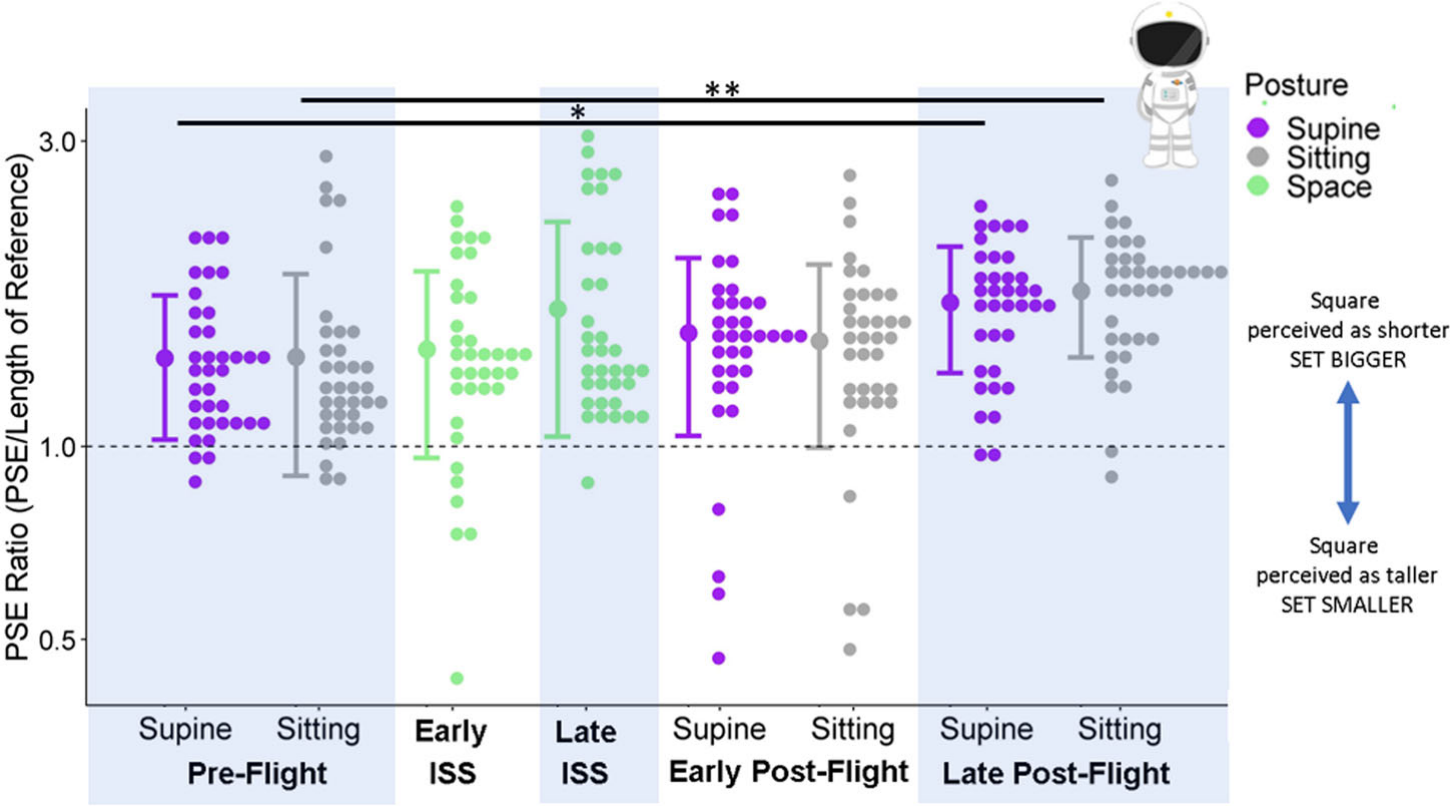
Accuracy in the Astronauts. Astronauts’ PSE ratios (relative to the reference stick) are plotted as histograms (little dots: distributions drawn at a bin width of 0.033 using the R package ggplot2^44^) on a logged y-axis for each session and posture (x-axis). The different postures are color-coded (purple for supine, grey for sitting, green for in space, and the dashed line indicate accurate performance. The large dots to the left of each distribution illustrate the mean ratio across all participants for a given session and posture, and the error bars are ± 1 standard deviation. One asterisk indicates a difference for which the 95% confidence interval did not include 0; two asterisks stars means that this effect was also significantly greater than in the control group as assessed by an interaction between the effect and the cohort (astronauts vs. controls).

See Fig. 1, and Supplementary Fig. 5A in Supplementary Materials C for a similar plot but broken down by distance of presentation.

We addressed the possibility of order effects, i.e., that participants learned or adapted to the stimulus in two ways: we repeated the same analysis, restricting it to those participants who did Supine as first posture in their pre-flight, or to those participants who did Sitting as first posture in their pre-flight session. Both analyses were compatible with the findings reported for our main analysis. We also tested within the pre-flight test session whether the order of testing had a significant influence on PSEs (i.e., whether the performance was different for the first posture they completed versus the second posture), which we found not to be the case.

#### Precision

**Fig. 2:**
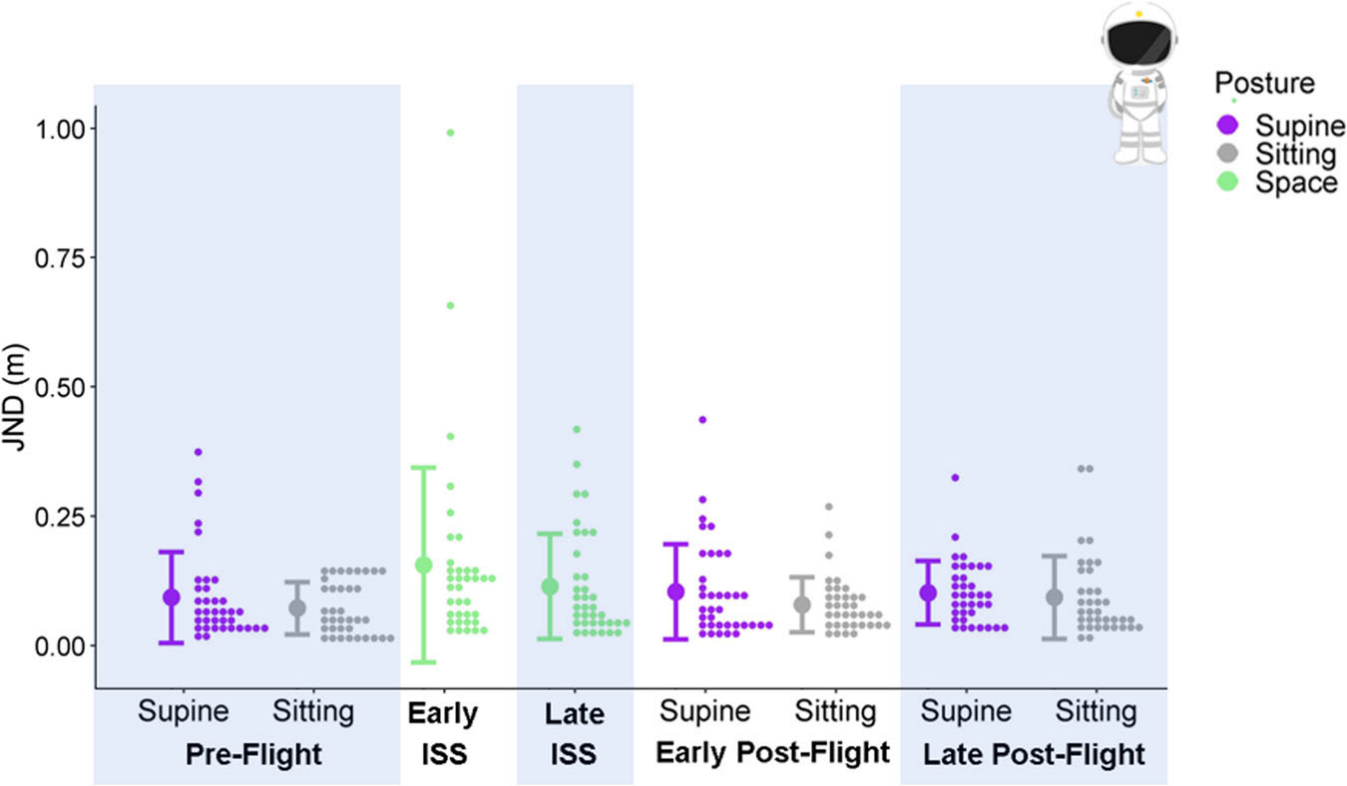
Precision in the Astronauts. Astronauts’ JNDs are plotted as histograms (little dots: distributions drawn at a bin width of 0.075) for each session and posture (x-axis). The different postures are color-coded (purple for supine, grey for sitting, green for in-space). The large dots to the left of each distribution illustrate the mean JND across all participants for a given session and posture, and the error bars are ± 1 standard deviation.

We found no differences in the astronauts’ JNDs for any of the contrasts we tested (see Fig. 2).

### Controls

#### Accuracy

**Fig. 3:**
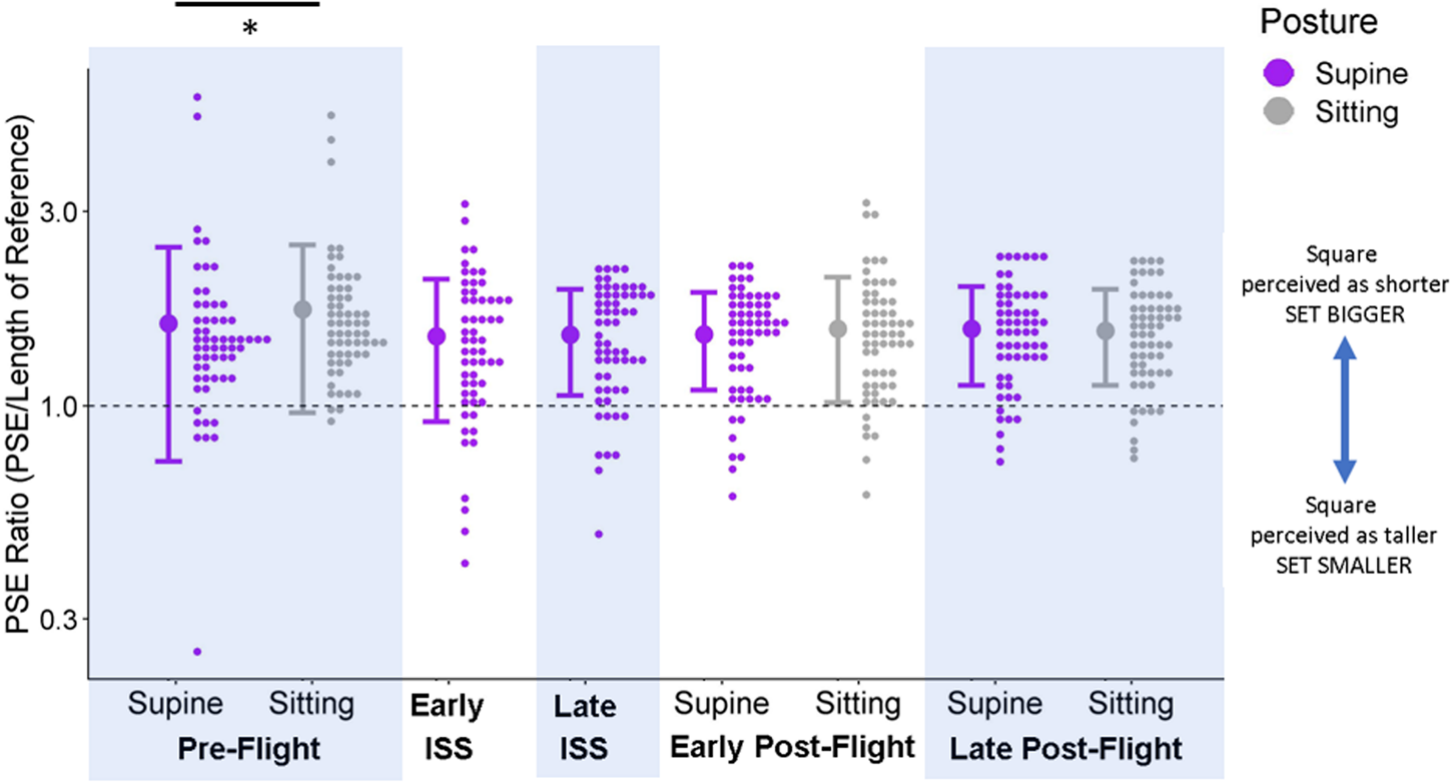
Accuracy in the Controls. Control participants’ PSE ratios (relative to the reference stick) are plotted as histograms (little dots: distributions drawn at a bin width of 0.033 using the R package ggplot2^44^) on a logged y-axis for each session and posture (x-axis). The different postures are color-coded (purple for supine, grey for sitting, and the dashed line indicates accurate performance. The large dots to the left of each distribution illustrate the mean ratio across all participants for a given session and posture, and the error bars are ± 1 standard deviation.

The accuracy model we used for assessing accuracy performance in the controls had an intercept of 1.74 (95% CI = [1.42;2.03]). We found that Supine elicited significantly lower ratios than Sitting at Pre-Flight (by 0.12, 95% CI = [0.03; 0.20]), which was not the case for Early Post-Flight and Late Post-Flight. No other difference contrasts were significantly different from zero. See Fig. 3, and Supplementary Fig. 5B in Supplementary Materials C for a similar plot but broken down by distance.

#### Precision

**Fig. 4:**
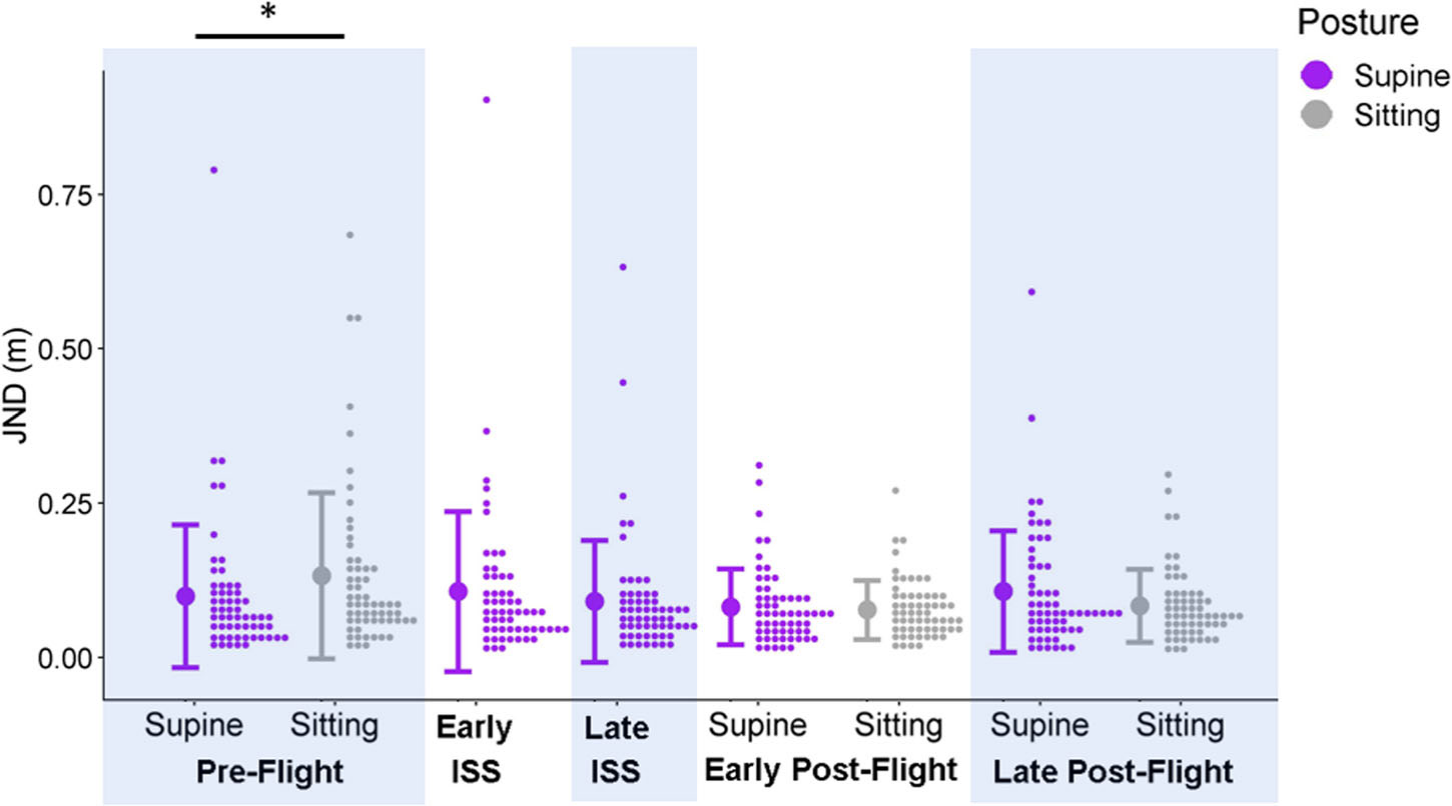
Precision in the Controls. Control participants’ JNDs plotted as histograms (little dots: distributions drawn at a bin width of 0.075) for each session and posture (x-axis). The different postures are color-coded (purple for supine, grey for sitting). The large dots to the left of each distribution illustrate the mean JND across all participants for a given session and posture, and the error bars are ± 1 standard deviation.

We found that JNDs were significantly lower for Supine than for Sitting at Pre-Flight (by 0.06, 95% CI = [0.03;0.08]), but not at Early Post-Flight or Late Post-Flight session. See Fig. 4.

We further conducted some exploratory analyses on differences between the men and women in our two cohorts, which are reported in Supplementary Material A.

## Discussion

For all sessions and for both our cohorts, the PSE ratios obtained were consistently set larger than 1, that is, the square had to be set larger to appear the same size as the reference stick. This was likely partially due to the well-documented tendency to compress space in VR^25^. We found no support for the hypothesis that altering the relationship between the gravity vector and the body(either by disrupting normal gravity perception or in the supine posture) could make the reference frame less reliable and thus lead to lower precision in object height judgements: for the astronauts, microgravity exposure did not increase JNDs relative to the on-Earth test sessions and no differences were observed between sitting and supine posture. For the controls, we found *decreased* JNDs for lying supine (the opposite of what we predicted) for the Pre-Flight session relative to when sitting upright, but this difference was no longer present in later sessions. Regarding accuracy, we observed significantly higher PSE ratios Late Post-Flight (60 days or longer after return) than at Pre-Flight or at Early ISS, a difference that we confirmed to be significantly larger than in the control group for the sitting posture but not for the lying supine posture. While, as apparent from Fig. 1, mean PSE ratios were about as high for Late In-Flight as for Late Post-Flight, examining the individual data points reveals that the mean is likely unduly influenced by several large data points, which at the same increased variability, widening the distribution. Controls displayed lower PSE ratios when lying supine than when sitting upright at Pre-Flight, again the opposite of what we predicted. This difference was no longer present as such Early or Late Post-Flight. An important caveat when interpreting our data is that we were not able to test astronauts in space until a few days after their arrival at the ISS (see Supplementary Table 1 in Supplementary Material B) and were similarly not able to test them immediately after their return to Earth. This leaves open the possibility that changes may have occurred from which the astronauts had adapted before we were able to test them.

A number of previous studies found changes in size and/or distance perception related to exposure to microgravity^5,7–10,17^. For example, Clément, Skinner & Lathan^6^ had participants adjust the dimensions of a box such that they perceived it as a cube. They found that, after about 90 days in microgravity, participants set a cube to be shorter and wider than on Earth, i.e., they perceived it as skinnier. Similar results have been reported during short-duration microgravity exposure during parabolic flights^5^. Contrary to these findings, we did not detect any statistically significant differences between the pre-flight test session and either of the two test sessions onboard the ISS. A relevant difference to our experiment here is that for the cube experiments^6^, the test stimulus was fairly close to the observer (at a distance of 50 cm), while our objects were simulated much further away (at 6, 12, and 18 m). Furthermore, our participants knew that the object in question was a square, so they could have used the width of the square to make their judgments rather than its height. They were, however, specifically instructed to focus on the height of the square, which makes this strategy somewhat less likely. Similarly, results showing that cubes drawn in weightlessness were drawn as smaller than on Earth^6,8^ were obtained within the astronauts’ peri-personal space suggesting that these results might not generalize to larger distances. Changes in perceived eye height (such as occur in microgravity^11^) have a large geometric effect on the perceived height of close objects but much less so for objects beyond a few meters^26^. Given the numerically large but non-significant difference between Pre-Flight and Late ISS, we further want to emphasize that our results do not allow the definite conclusion that microgravity left size judgments unaffected; the lack of significance may be simply due to a lack of power (e.g., due to a higher-than-expected variability). Finally, a recent study by Morfoisse et al.^27^ suggests that gravity has a similar effect on haptic perception as on visual perception. If, indeed, our participants’ perception of the reference length was affected by gravity in a similar way as their perception of the visual target, this may explain an absence of significant differences in our experiment.

One surprising finding in our study was changes in size perception in our astronauts that appeared only long after return to Earth: the target size was set much larger in the last test session (Late Post-Flight – at least 60 days after return) than it had been set in earlier test sessions, indicating that they underestimated its height more than they had earlier. Importantly, this difference cannot be explained by learning effects or a loss of interest in performing the experiment properly. Firstly, both learning and loss of interest should affect precision, not accuracy. Secondly, control participants’ performance remained stable across all five test sessions. While to our knowledge no long-term consequences of microgravity exposure have been reported for the perception of size or distance, Harris et al.^28^ did report reemerging changes in the perception of orientation in astronauts months after the return. A potentially pertinent observation, which may be in line with this result, is that PSEs tended to increase over the five test sessions for the astronauts but not for the controls. Such a trend might indicate the perceptual apparatus becoming more malleable. It is, however, not immediately clear why – in the absence of feedback of any kind – such changes in accuracy might occur. A further potential explanation might be that increased noise in the vestibular system during microgravity exposure might lead to a perceptual strategy that disregards the multisensory cues which, under regular gravitational conditions, allow scaling of the environment. A learned neglect of such cues might then lead to a “flattening” of visual space that persists after a return to normal gravitational conditions (in the Late Post Flight session).

Another important goal of this study was to confirm and expand the results of previous studies^12,13^ that had reported needing to set the target larger while supine than while upright. However, contrary to these earlier findings, our participants either set the targets *smaller* while supine compared to when upright (the controls at the Pre-Flight session) – the opposite direction of the effect of previous studies – or we found *no difference* between sitting and lying at all (the remaining control test sessions and in the astronaut cohort). This is in stark contrast with previous results: Harris and Mander^12^ found that a projected line needed to be made 9% longer when supine than when sitting upright to match a reference stick held in their hands. Similar results were also reported by Kim and Harris^13^ using a stereo virtual reality display that provided disparity cues where participants needed to make the target 5.4% larger when supine and 10.1% larger when prone compared to when upright.

Conceptually, our study was very similar to the experiment conducted by Harris and Mander^12^; however, there were several differences: First, Harris and Mander^12^ used a real environment while we presented our stimuli in virtual reality through an Oculus head-mounted display that did not provide disparity cues of the scene. The overall size judgements in the Harris and Mander study were close to correct (~97% of the length of the reference stick) when upright and viewing monocularly, compared to our mean settings of 153% of the length of the reference stick, suggesting that size/distance judgements in a real environment were considerably more accurate in general than in the present study. Their participants also had a larger field-of-view not limited by the constraints of the field-of-view of an HMD. Finally, the distance to the target was much smaller (1.22 m and 3.66 m) than in the present study (6 m, 12 m, 18 m).

**Fig. 5:**
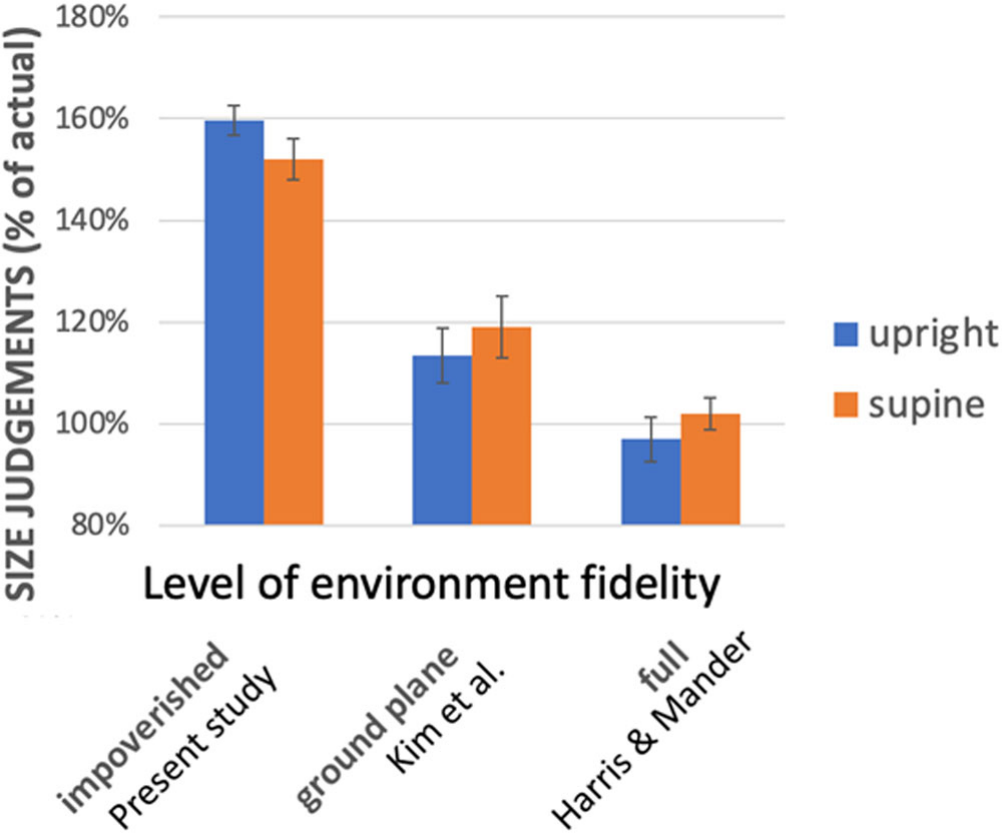
Comparison of Height Judgements Across Studies. Comparison of height judgments made in different environments in three separate studies. 100% indicates accurate performance. Note that setting the size as larger indicates they perceive it as smaller and need to compensate. Present study values from controls “pre-flight”.

A recent comparable study of the effect of posture on height perception by Kim et al.^13^ used virtual reality and reported overall PSEs of 119.8% of the reference stick. Unlike ours, this study used binocular cues and a more realistic simulated environment including a textured ground plane and an implied horizon but was performed, like the present study, using virtual reality. While the distances used were more similar to ours than Harris and Mander’s, they were still on average closer than ours (2, 5, 7, and 10 m). Our experiment was conducted using an HMD with no simulated view of the participant’s own body, while Harris and Mander’s^12^ participants (but not Kim et al.’s^13^) were able to see their body and the reference stick. Figure 5 plots height judgements for these three studies as a function of the type of environment used: the full, real-world environment from Harris and Mander^12^, the virtual environment with a textured ground plane from Kim et al.^13^ and the virtual environment with an untextured ground plane from the present study. More impoverished environments were associated with larger settings indicating the targets were perceived as smaller. These differences in design, however, do not provide an explanation for the different effects of posture found in these studies.

A potentially more impactful difference between the Harris and Mander^12^ study, the Kim et al.^13^ study, and the present study than the effect of the environment were that in none of those studies did the participants’ eye height change with posture. In Harris and Mander^12^ participants remained seated in the York Tumbling Room throughout the experiment. Their eye height on the raised chair was thus always the same distance above the floor at approximately normal standing height. Additionally, their stimulus was positioned directly in front of the participant so that eye height was not necessary to interpret the geometry of the visual stimulus as it would be for an object on the ground. Kim et al.’s^13^ participants stood during the upright condition and lay flat for the supine and prone conditions. Eye height is known to affect the perceived size of an object with a lower eye height making things appear smaller^29,30^ and may have contributed to Bourrelly et al.’s findings^10,11^ concerning the minimum width necessary to fit through an aperture. We further found in a recent study from our lab that eye-height affects perceived size differently depending on posture^26^. Although in our experiments the entire visual simulation including the simulated eye height above the floor of the corridor always remained fixed, the fact that the experiments (on Earth) were carried out physically sitting in a chair or lying supine stretched out on a bed may have contributed to the general absence of a difference between the sitting and supine postures. If our participants used their real-world seated eye height (around 1.2 m) as opposed to the simulated eye height of 1.85 m to scale the environment when seated, this may have contributed to participants interpreting the whole scene (including the square) as smaller (and thus needing to be set larger to match the reference) than when lying. Such an effect would tend to cancel the tendency to perceive the world as larger when upright (and thus needing to be set smaller) as reported by Harris and Mander^12^ and Kim et al.^13^. When lying supine, eye height would affect height judgments less because the real-world eye height is then ill-defined. And in fact, the recent data from our lab^26^ provide evidence consistent with this explanation: changes in simulated eye height indeed affect object height judgments more when participants sit upright than when they are lying supine.

**Fig. 6:**
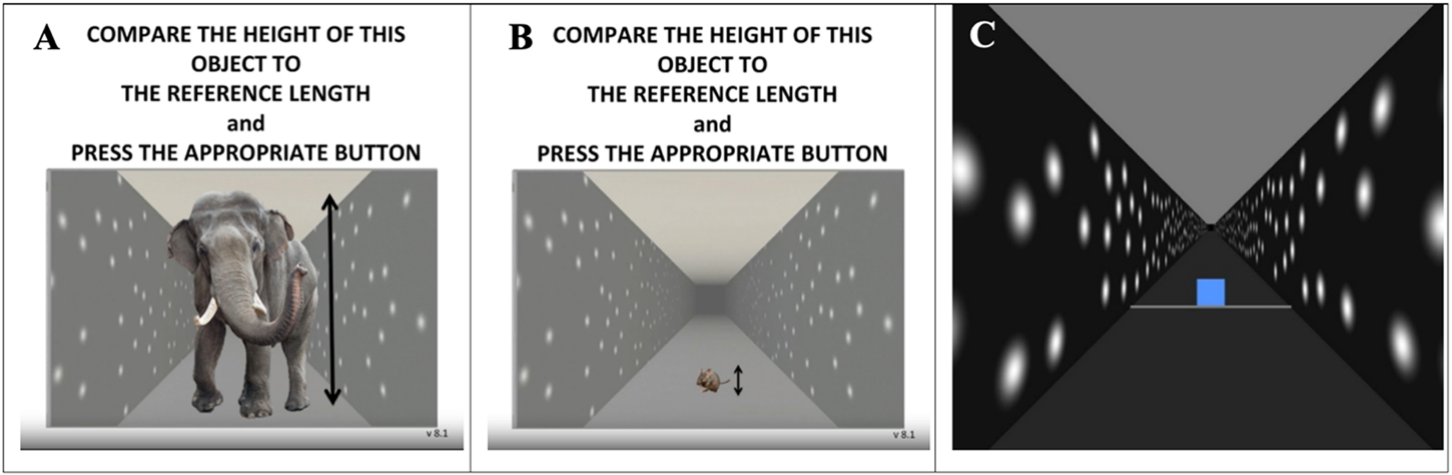
Screenshots from the Experiment. **A**–**C**.: Screenshots from the experiment. **A**, **B**: testers to make sure that participants use the correct buttons to respond to the stimulus. **C** Screenshot from the hallway in which the participants were immersed, along with a sample virtual 2D square the height of which they compared to the length of a reference stick held in their hands aligned with the long axis of their body.

We did not find the expected decrease in precision when lying supine relative to sitting upright, failing to support a direct vestibular effect on height judgments. In fact, the controls displayed an *increase* in precision when lying supine over sitting upright in one of the test sessions – a paradoxical finding given the absence of a theoretical foundation or supporting evidence in the literature. In the context of this null finding, it is important to note that JNDs may be underestimated when obtained through a PEST staircase procedure. We partially mitigated this by fitting psychometric functions to data from both staircases for each condition (one starting above and one starting below the expected PSE). Further, any such bias should affect all our experimental conditions equally and would therefore not be expected to have an impact when comparing between conditions. Any such effects in precision may have been too small to detect and clearly, from Harris and Mander^12^ and Kim et al.^13^, there is an effect of posture (Fig. 6) that can only be from a change in the relative direction of gravity on the body or the proprioceptive cues related to the sitting and lying supine postures. It is important in this context to note that we were not able to test any astronauts before their third day onboard the ISS by which time they may have already become acclimatized to their new gravitational environment – the same limitation applied when the astronauts returned to Earth (see Supplementary Table 1 in Supplementary Material B).

Overall, we found no evidence for the gravity-as-reference-frame hypothesis: few changes in precision were observed, and where there were differences, they went in the opposite direction of what would be predicted by this hypothesis. Further, we observed a postural effect that seemingly contradicts previous findings. However, differential effects of simulated eye height depending on posture may provide an explanation here. Finally, the astronaut cohort displayed late-appearing changes in object height perception, with a large underestimation at the last test session at least 60 days after return in comparison to earlier test sessions (Pre-Flight and Early ISS sessions, specifically).

These are positive findings for space flight safety, both for current astronauts and potential future commercial space flight operations. The absence of an effect of microgravity exposure during space flight on the perception of object height (at least after the first few days in space) indicates that tasks that rely on accurate and precise height judgments can be conducted safely fairly soon after arrival in space. Latent biases that emerge robustly several weeks after return to Earth may, however, be a reason for concern and should be included in briefings for commercial and non-commercial space travelers. Our data do not warrant strong conclusions concerning sex and/or gender differences: while the data were largely inconclusive, this at the minimum rules out stark differences between men and women that would require differential treatment of both groups during or after space flight.

## Methods

### Participants

Astronauts – Fifteen astronauts completed the first test session on Earth. One was excluded because they could not complete the first on-orbit test session within the time window (within 6 days after arrival on orbit), and the space flights of two others were delayed until after the full data set of 6 women and 6 men had been collected, i.e., no microgravity or post-flight data were collected from them. The remaining twelve astronauts (6 women and 6 men) were on average 42.6 years old (SD = 5.4 years), with a mean age of 38.7 years for the women and 46.6 years for the men. See Supplementary Table 1 in Supplementary Materials B for the timing of the testing sessions.

Controls – 22 control participants were enrolled in this experiment. One participant dropped out after their first session, and another during their second session. Both partial data sets were excluded from the analysis and the remaining 20 participants (10 women and 10 men) were 42.6 years old (SD = 7.2 years), with a mean of 43.9 years for women and 41.3 years for men. See Table B2 for the timing of the testing sessions.

All participants reported normal or corrected-to-normal vision and reported no vestibular issues, nor problems with balance or depth perception. Control participants were compensated for their travel costs to the site of data collection. The research was approved by the ethics committee of York University as well as the ethics boards of NASA and the relevant space agencies of the region of origin of each astronaut participant. All participants read and signed an informed consent form. The experiment was conducted in accordance with the Code of Ethics of the World Medical Association (Declaration of Helsinki, 1964).

### Apparatus

We used an Oculus Rift CV1 (Oculus VR, Menlo Park, USA) virtual headset with a diagonal visual field of about 110° to present the stimuli. This HMD has a resolution of 1080 × 113041998@Gma200 pixels per eye and a refresh rate of 90 Hz. Stimuli were programmed in Unity (2017.1.0f3) and the display in the HMD was presented as head-fixed. Both eyes were presented with the same image such that no disparity cues to scene depth were present. We used an HP IDS DSC 4D Z15 Notebook PC (Hewlett-Packard, Palo Alto, USA) with an Intel Core i7-4810MQ Quad Core processor (Intel Corporation, Santa Clara, USA) and an NVIDIA Quadro K610M graphics card (NVIDIA Corporation, Santa Clara, USA) to generate the display and control the experiment. All responses were given by means of a 3G Green Globe Co Ltd (FDM-G62 P) finger mouse (3G GREEN GLOBE, Taipei, Taiwan). A steel stick (38.1 cm long, 2.5 cm wide, 4 mm thick) with smoothed corners was used as a reference stick. Participants wore a cervical neck collar (Optec Proglide Cervical Collar Neck Brace) to minimize head movements relative to their body.

### Stimuli and Procedure

Participants were immersed in a virtual reality hallway environment (Fig. 6C) that extended out in front of them while they were either seated upright or lying supine stretched out on a bed. The virtual environment was head-fixed such that the visual stimulation was always in a fixed orientation relative to their body. The hallway was simulated as 3.3 m high and 3.3 m wide with a dark floor and lighter ceiling, and an implied horizon. The simulated viewpoint for rendering was from the center of the corridor corresponding to a constant simulated eye height of 1.65 m, that is, in the middle of the simulated hallway. Participants were told to imagine themselves standing on the floor surface. White Gaussian blobs (diameter = 0.8 m, sigma= 0.2 m) were presented at random locations on the wall to strengthen the perspective cues.

On each trial, a blue 2D square was presented at one of three simulated target distances (8, 12, and 16 m) in front of the participant. A white line is drawn on the floor aligned with the square to help ground the square on the otherwise featureless visual floor. Participants were asked to judge whether the height of the square was taller or shorter than the length of an external reference stick (38.1 cm) that they held aligned with the long axis of their body with one hand at each end. The specific instructions participants received were “You must determine if the side of the square on the screen is smaller than the reference stick, or if the side of the square on the screen is taller than the reference stick”, that is, their attention was drawn to the height of the square. The reference stick was held parallel to the long axis of their body with one hand on the upper end of the stick and the other on the lower end. Participants saw this reference stick briefly while receiving the instructions, but they had no view of the reference stick while making these judgments, and no reference stick was simulated in the VR environment. Thus, participants had a primarily haptic estimate of the length of the reference stick. Participants indicated their decision by clicking a mouse held in their chosen hand – left-click if the height of the square appeared shorter than the reference stick or right-click if it appeared taller. Before starting the first trial, participants rehearsed the task in a practice test using a very large (an elephant) and a very small (a mouse) simulated object (Fig. 6A, B) to ensure they correctly understood which button indicated which decision. After successfully completing the test, they began the first trial. The instructions were presented in the HMD throughout the experiment, which included an image of a person standing in the hallway as a visual aid. On Earth, the instructions were also read out to the participants during each session, while in space they had Standard Operating Procedures available to them and we provided them with a “BIG PICTURE” document that briefly repeated all important information about the experiment for them to consult prior to or during their ISS test sessions.

The horizontal and vertical dimensions of the projected 2D square (to keep it a square) were adjusted under the control of Parameter Estimation Sequential Testing (PEST) adaptive staircases^31^ that each terminated after 25 trials or after 13 reversals (i.e., when two subsequent perceptual decisions on any given staircase were different), whichever came first. Two staircases were performed for each of the three target distances, with one starting with a side length double the length of the reference stick (76 cm) and the other starting with a side length of half of the reference stick’s length (19 cm) for a total of six randomly interleaved staircases per posture and session. Lower and upper bounds were set for the staircases at 9.5 cm and 228 cm respectively. A video of the task can be found on the Open Science Foundation website: https://osf.io/t47nd.

The experiments reported here are part of a suite of three experiments that we performed on the ISS. One intended to simulate visual gravity through accelerated visual self-motion. Participants experienced a period of sideways visual self-motion, after which they adjusted a virtual ground plane to match their perception of their orientation. This experiment has as of publication of the current manuscript not been published. Our other experiment^32^ investigated the perception of traveled distance. Participants saw a target ahead of them. Upon mouse click, it disappeared, and they experienced optic flow consistent with forward self-motion. Then, they pressed a button when they thought they had traveled the distance to the target. These three experiments were always performed in the same test sessions; first the orientation judgments, then the judgments on traveled distance, and finally the task reported in this manuscript. No other experiments were consistently performed before these test sessions, and a requirement was in place that no experiments affecting visual or vestibular function (e.g., involving medication affecting either of the systems) be scheduled in the 24 hours before our experiment.

### Test sessions

For both astronauts and controls, there were five test sessions carried out over the span of about one year (see Supplementary Materials B for the detailed timing of each test). The astronauts were tested once before their space flight (Pre-Flight), upon arrival on the International Space Station (Early ISS; between 2 and 6 days after arrival), after about 60 days in space (Late ISS), then after return to Earth (Early Post-Flight; within 7 days of return) and about 90 days after the return (Late Post-Flight). During Pre-Flight, Early Post-Flight, and Late Post-Flight test sessions, participants completed the experiment in two different body postures: sitting upright and supine. The order of postures was counter-balanced across participants and test sessions. In the Early ISS and Late ISS sessions, the astronauts were floating freely, with a backrest loosely attached to the deck preventing them from drifting away from the test area onboard the ISS.

Controls followed the same sequence of test sessions at similar intervals, the only difference being that they performed the Early ISS and Late ISS sessions lying supine on Earth. These correspond to the two test sessions the astronauts completed onboard the ISS. For the controls, the second test session occurred on average 84 days after the first test session (SD = 39 days). The third session occurred 173 days (SD = 40 days) after the first one, the fourth one 302 days (SD = 40 days) after the first one, and the fifth one 359 days (SD = 41 days) after the first one.

### Data analysis

#### Fitting of psychometric functions

We used the R^33^ package quickpsy^34^ to fit psychometric functions to the full staircase data for each participant, session, posture, and target distance separately. quickpsy fits means and standard deviations of cumulative Gaussian functions by direct likelihood maximization^35,36^. For optimization, we opted for the Differential Evolution algorithm^37^. This algorithm requires upper and lower bounds for the parameters; we chose the minimum and maximum height presented by the staircase (9.5 cm and 228 cm) for the means and 0 - 500 cm (which corresponds to Weber Fractions well above any expected for height judgements^38,39^) for the standard deviation. The fitted standard deviations of these psychometric functions are equivalent to the 84.1% Just Noticeable Difference (JND), which is a measure of precision, with higher JNDs corresponding to lower precision. The means correspond to the Points of Subjective Equality (PSEs), which represent a measure of accuracy.

#### Outlier analysis

We excluded the data from all conditions associated with sessions where participants responded in such a way that, for a given PEST, five or more of the presented target heights were at the upper or lower bounds (i.e., 9.5 or 228 cm). Such conditions were excluded as they corresponded to unreasonable responses (target being unreasonably small or tall). This criterion led to the exclusion of the entire second session (which was conducted in the supine posture) from one female control participant and the supine posture from each of one male and one female control participant from their first session. No sessions were excluded from any of the astronauts. Statistics were performed using R v3.6.1.

#### Data processing

For reporting, we converted the PSEs into ratios by dividing them by the actual length of the reference stick:

1 $${\rm{PSE\; Ratio}}={\rm{PSE}}/({\rm{Actual\; reference\; length}})$$

Ratios larger than 1 indicated that a given PSE was larger than the reference stick (corresponding to compensating for an underestimation of the perceived height of the square), and ratios below 1 indicate that the PSE was smaller than the reference stick (corresponding to an overestimation of the perceived height of the square).

We did not pre-process the JNDs in any way.

#### Linear mixed modeling

We employed linear mixed modeling (LMM) using the lme4 package^40,41^ for R. When choosing the structure of these statistical models, following standard techniques^42^ we opted to use the independent variable(s) of interest as fixed effect(s), while representing all other known sources of variability as random effects.

Since we were interested in the main effects of the test session, posture, and their interaction on PSEs and JNDs, we used Session (a discrete variable with the levels “Pre-Flight”, “Early ISS”, “Late ISS”, “Early Post-Flight”, “Late Post-Flight”), Posture (“Sitting” and “Supine” for terrestrial postures and “Microgravity” for the astronauts in “Early ISS” and “Late ISS” sessions only), their interaction, and Target Distance (as a categorical variable with the values 8 m, 12 m, and 16 m) as fixed effects and completed the model^42^ with random intercepts as well as random slopes for Session, Posture, and Distance per Participant as random effects. We fitted two models, one for accuracy and one for precision. For accuracy, we used the fitted PSE Ratios as dependent variables, while we used the fitted JNDs as the dependent variable to test for precision differences. For precision, since higher PSEs are generally related to higher JNDs, we also added the PSE ratios as a fixed effect because we were interested to what extent Posture and Session explained variability beyond their immediate influence on accuracy. The Wilkinson and Rogers^43^ formalisms for the two models described in this paragraph are:

2 $$\begin{array}{l}{\rm{PSE}}\; {\rm{Ratio}} \sim {\rm{Session}}* {\rm{Posture}}+{\rm{Target}}\; {\rm{Distance}}\\+(1+{\rm{Posture}}+{\rm{Target}}\; {\rm{Distance}}+{\rm{Session}}|{\rm{Participant}})\end{array}$$

3 $$\begin{array}{ll}{\rm{JND}} \sim {\rm{Ratio}}+{\rm{Session}}* {\rm{Posture}}+{\rm{Target}}\; {\rm{Distance}}\\+\,\left(1+{\rm{Posture}}+{\rm{Target}}\; {\rm{Distance}}+{\rm{Session}}|{\rm{Participant}}\right)\end{array}$$

For Null Hypothesis Significance Testing, we computed confidence intervals for the fixed effect coefficients using a bootstrap method, implemented in the confint function from the R stats package^33^. When the confidence interval did not include zero, we concluded that the independent variable was significantly related to the dependent variable. All code for analysis, as well as the anonymized raw data and the fitted PSEs can be found in the following Open Science Foundation repository: https://osf.io/wvg9z/. Terms starting with a capital letter refer to variables in these models.

Wherever we found significant differences in the astronaut group we confirmed whether such effects were dissimilar from those found in the control group (which might be caused, for example, by learning) by assessing the interaction between the cohort (astronauts vs. controls) and the effect in question. In order to avoid a triple interaction, we conducted this further analysis only over the relevant subset of the data. A dummy specification for this test in the Wilkinson & Rogers formalism is: in Equation 4, PSE and JND should be separated by either a back slash (/) or the word OR. This is not meant to be a fraction.

4 $$\begin{array}{l}\\{PSE/JND} \sim {\rm{Cohort}}* {\rm{Effect}}+{\rm{Target}}\; {\rm{Distance}}\\+\left(1+{\rm{Effect}}+{\rm{Target}}\; {\rm{Distance}}|{\rm{Participant}}\right)\end{array}$$

A significant interaction (as assessed using bootstrapped confidence intervals as above) would then mean that the effect was stronger in the astronaut cohort than in the control cohort.

We performed the following set of planned comparisons to test our a priori hypotheses:

- Astronauts
  ○ Pre-Flight (both sitting and lying) will have lower PSE’s and JND’s than Early ISS and Late ISS (to test for an effect of microgravity).
  ○ Pre-Flight (both sitting and lying) will have lower PSE’s and JND’s than Early Post-Flight and Late Post-Flight (to test for recovery to baseline after microgravity exposure).
  ○ Sitting will have lower PSE’s and JND’s than Lying at Pre-Flight, Early Post-Flight and Late Post-Flight.
- Controls
  ○ Sitting will have lower PSE’s and JND’s than Lying at Pre-Flight, Early Post-Flight, and Late Post-Flight.
- For any effects in the astronauts: interaction between the effect and the cohort type (controls versus astronauts).

## Supplementary information

Supplementary Materials

## Data Availability

The data can be found on Open Science Foundation (https://osf.io/wvg9z/).

## Code availability

The code used for analysis can be found on Open Science Foundation (https://osf.io/wvg9z/).
