# Supplementary material for: The impact of gravity on perceived object height
Source: NPJ Microgravity. 2024 Oct 4;10:95. doi: 10.1038/s41526-024-00430-3 (PMC11452668; doi:10.1038/s41526-024-00430-3)
Supplement: Supplementary file 1 — Supplementary Materials [file 41526_2024_430_MOESM1_ESM.pdf]

The Impact of Gravity on Perceived Object Height – Supplementary Materials

Björn Jörges[1]*, Nils Bury[1 2], Meaghan McManus[1 3], Ambika Bansal[1], Robert S. Allison[1], Michael Jenkin[1], Laurence R. Harris[1]

[1]Center for Vision Research, York University, 4700 Keele Street, Toronto, ON M3J 1P3, Canada

[2]Institute of Visual Computing, Hochschule Bonn-Rhein-Sieg, Grantham-Allee 20, 53757 St. Augustin, Germany

[3]Department of Experimental Psychology, Justus Liebig University Giessen, Otto-Behaghel-Strasse 10F, 35394 Giessen, Germany

*Corresponding author

Keywords

Perceived size, perceived height, microgravity, posture, virtual reality

Supplementary Materials A – Sex/Gender Differences

One of the stated goals of this project was to explore whether there were any sex and/or gender differences in performance in general, or in any of the effects we found in this study, and whether such differences might impact performance in microgravity in a way that might require differential safety precautions for women and men.

The idea that there might be gender-related differences in size perception is based on reported differences between women and men in visual[1,2], vestibular[3,4] and visuo-vestibular[5] tasks as well as the relative prevalence of vestibular disorders[6] in women.

Analysis – Given the sample size that is fairly small for the detection of any between-participants effects and given that we were also interested in quantifying evidence in favor of the null hypothesis where relevant, we used Bayesian Linear Mixed Modelling as implemented in the brms package for R[7] for this analysis. We fitted separate models for accuracy and precision and astronauts and controls respectively. Their structure followed the models we fitted for the main analysis in this paper, except that we added sex/gender as well as all interactions between sex/gender and Posture and Session. Since we had no strong prior expectations, we used shallow priors for all relevant effects. The Wilkinson & Rogers notations for our models were the following:

Ratio ~ Session*Posture*Gender + (1 + Posture + Target Distance + Session | Participant) (1)

JND ~ Ratio + Session*Posture*Gender + (1 + Posture + Target Distance + Session | Participant) (2)

We then computed Bayes Factors using the Savage-Dickey density ratio method as implemented in the `hypothesis` function from brms in order to assess to what extent the data increased our confidence in the specified hypothesis for any given effect. By default, the point hypothesis will be the absence of a difference between women and men.

Results – For the accuracy data in the astronauts (see Supplementary Figure 1), we found a Bayes Factor of 1.88 (indicating anecdotal evidence as per Andraszewicz[8]) for an absence of overall performance differences between women and men. We further observed Bayes Factors of 1.2 (Early ISS) and 1.19 (Late ISS) for the interaction between Sex/Gender and Test Session, indicating inconclusive evidence for the hypothesis that women and men reacted equally to microgravity exposure. Our data lastly provided anecdotal evidence that women and men reacted equally to the postural manipulation (with a Bayes Factor of 2.55).

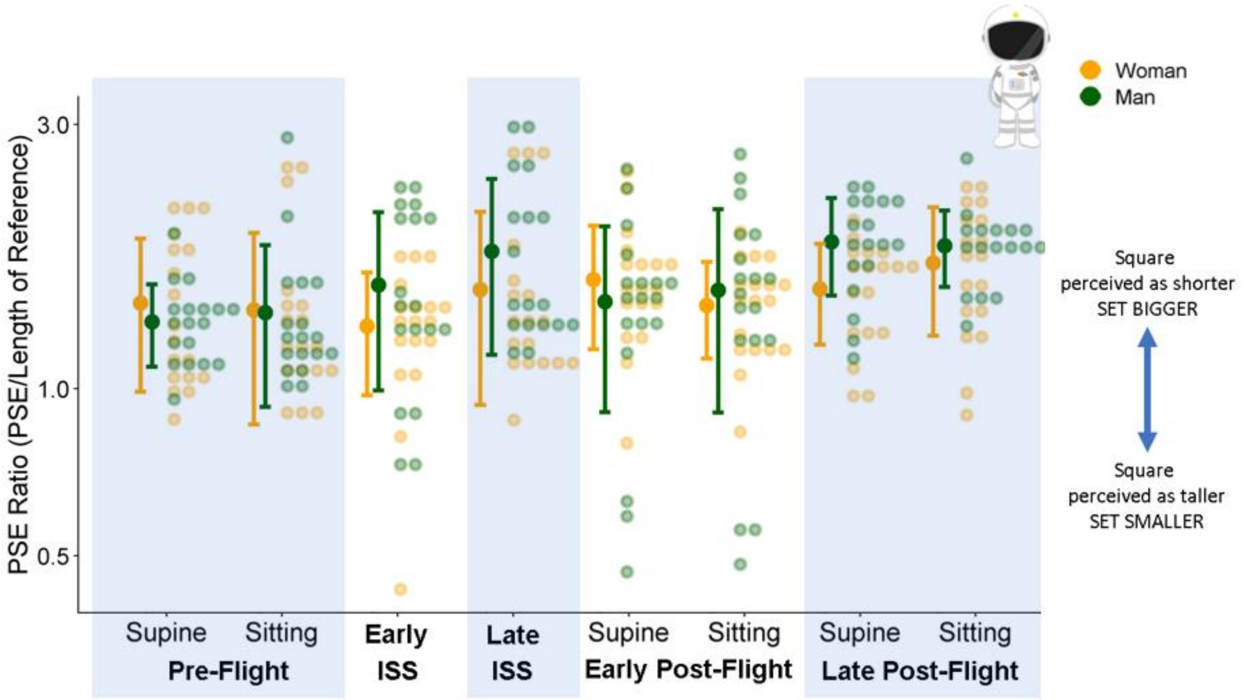

Supplementary Figure 1: Astronauts' PSE ratios plotted as histograms (little dots; distributions drawn at a bin width of 0.033) on a logged y-axis for each session and posture (x-axis). The sex/gender of the participants is color-coded (yellow for women, green for men), large dots to the left of the distributions illustrate the mean ratio across all participants for a given session and posture, and the error bars are ± 1 standard deviation.

Regarding precision (see Supplementary Figure 2), we found compelling evidence that women and men performed equally overall (with a Bayes Factor of 10.11). When testing for whether women and men reacted differently to microgravity exposure, we found again inconclusive evidence with Bayes Factors of 1.2 (Space 1) and 1.26 (Space 2) respectively. Finally, there was moderate evidence (Bayes Factor of 7.73) that men's and women's precision was affected equally by the postural manipulation.

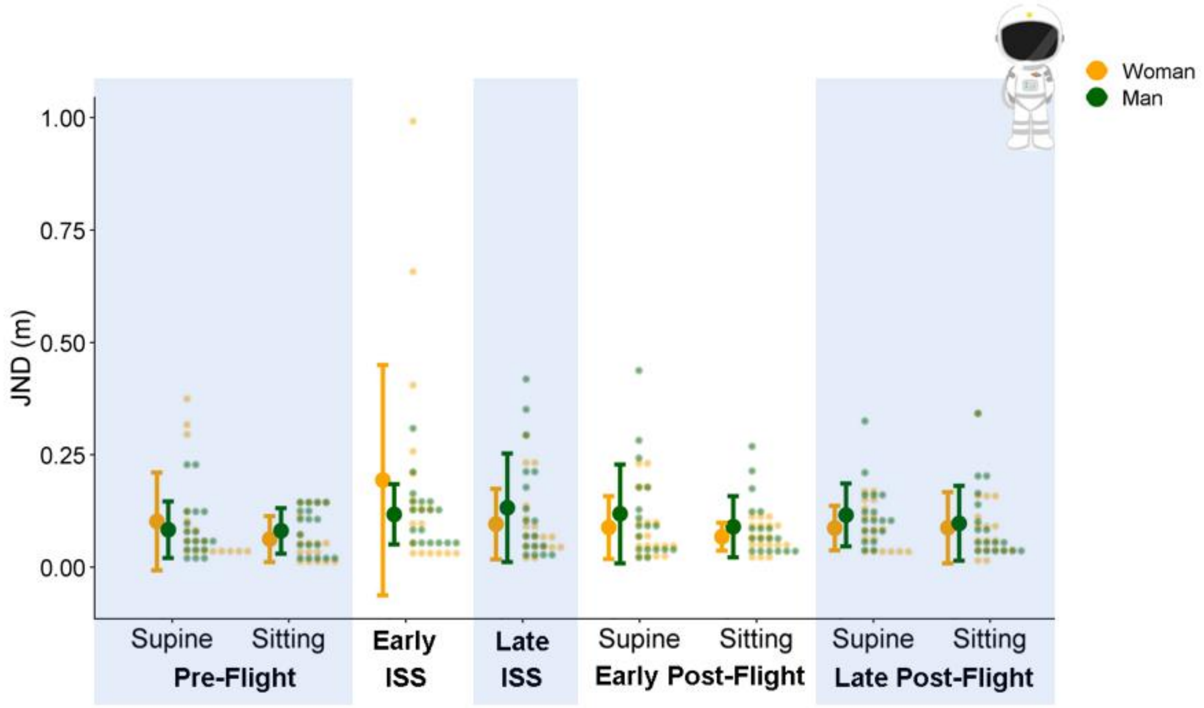

Supplementary Figure 2: Astronauts' JNDs plotted as histograms (little dots; distributions drawn at a bin width of 0.075) for each session and posture (x-axis). The sex/gender of the participants is color-coded (yellow for women, green for men). The large dots to the left of the distributions illustrate the mean JND across all participants for a given session and posture, and the error bars are ± 1 standard deviation.

In the controls, we observed a Bayes Factor of 1.99 (indicating anecdotal evidence) in favor of there being no overall difference between women and men in accuracy (see Supplementary Figure 3), while we found moderate evidence (Bayes Factor of 3.86) that male and female controls were impacted similarly by the postural manipulation.

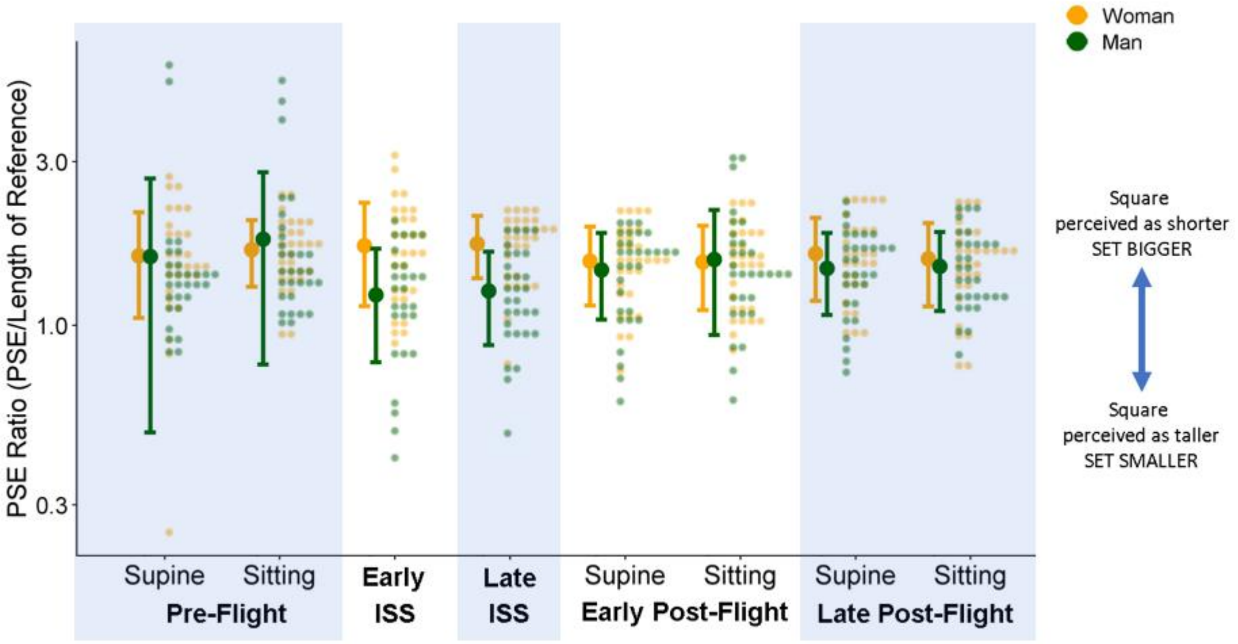

Supplementary Figure 3: Control participants' PSE ratios plotted as histograms (little dots; drawn at a bin width of 0.05) on a logged y-axis for each session and posture (x-axis). The sex/gender of the participants is colour-coded (yellow for women, green for men). The large dots to the left of the distributions illustrate the mean ratio across all participants for a given session and posture, and the error bars are ± 1 standard deviation.

In terms of precision (see Supplementary Figure 4

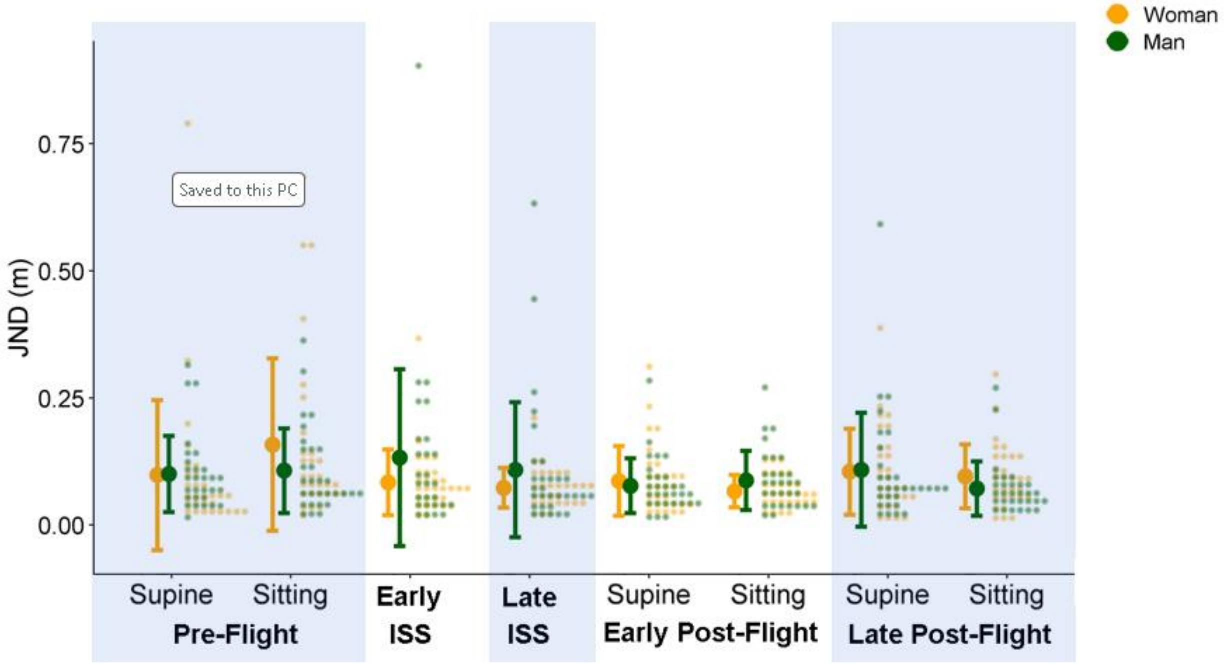

), we found moderate evidence both against an overall difference between women and men (Bayes Factor of 4.06) and against the hypothesis that women and men reacted differently to the postural manipulation (Bayes Factor of 3.28).

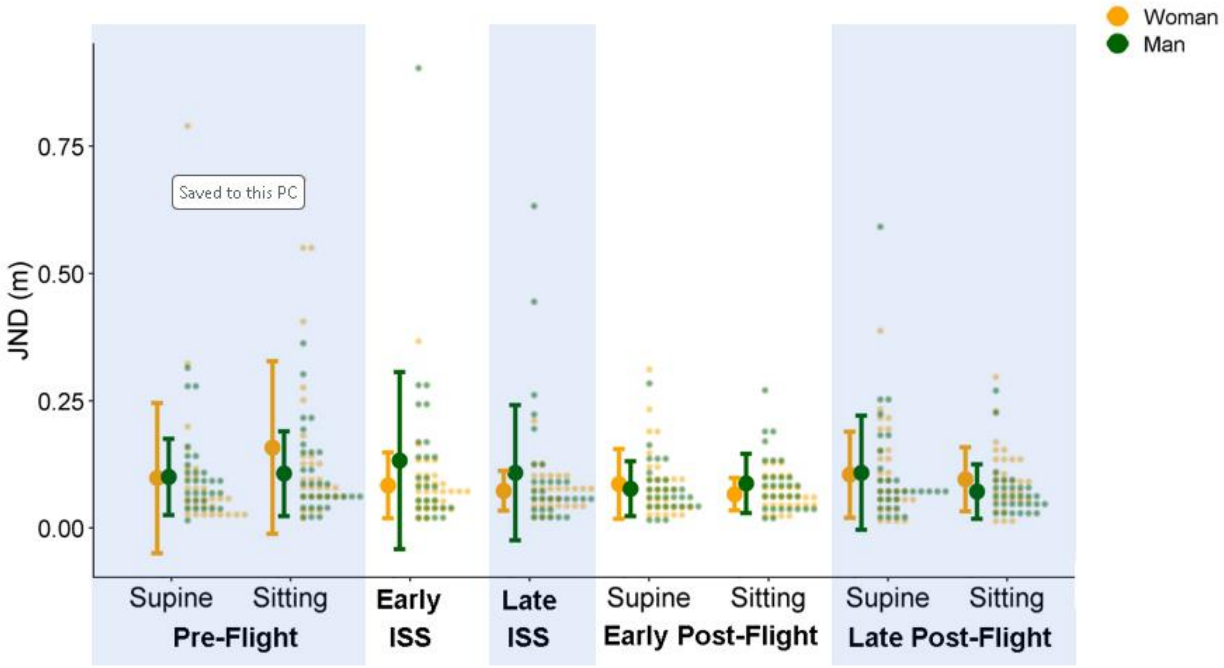

Supplementary Figure 4: Control's JNDs plotted as histograms (little dots; distributions drawn at a bin width of 0.075) for each session and posture (x-axis). The sex/gender of the participants is color-coded (yellow for women, green for men). The large dots to the left of the distributions illustrate the mean JND across all participants for a given session and posture, and the error bars are ± 1 standard deviation.

Discussion – Overall, we found anecdotal to moderate evidence against there being differences between women and men, both in terms of accuracy and precision. While this evidence is far from conclusive, it suggests that female and male astronauts are likely served well by a similar set of precautions, safety protocols, and technological aids when it comes to tasks that heavily rely on accurate and precise size and/or distance perception.

Supplementary Materials B – Participant sessions

Supplementary Table 1: The timing of each test session (BDCs and on-orbit test sessions) for each astronaut relative to launch (L) and return (R), along with aggregate statistics (mean and standard deviation) of the time between the test session and launch.

#	Gender	Pre-Flight	Early ISS	Late ISS	Early Post-Flight	Late Post-Flight	Total Time In Space
1	M	L-87	L+3	L+86	R+6	R+62	204
2	F	L-87	L+3	L+86	R+6	R+62	204
3	F	L-183	L+5	L+95	R+6	R+180	329
4	M	L -186	L+5	L+87	R+6	R+124	201
5	M	L-184	L+5	L+87	R+6	R+54	272
6	M	L-204	L+4	L+92	R+6	R+62	196
7	F	L-111	L+6	L+92	R+4	R+54	185
8	M	L-264	L+3	L+85	R+5	R+46	167
9	M	L-165	L+3	L+83	R+3	R+57	200
10	F	L-147	L+3	L+84	R+4	R+165	176
11	F	L-247	L+3	L+83	R+6	R+61	170
12	F	L-141	L+3	L+83	R+4	R+87	170
	**#M: 6 #F: 6**	**Ø: L-167d ±56.5d**	**Ø: L+3.8d ±1.1d**	**Ø: L+86.9d ±4d**	**Ø: R+5.1d ±1.1d**	**Ø: R+84.5d ±46d**	**Ø: 206.2d ±47.8d**

Supplementary Table 2: This table gives an overview of our schedule for the participants of our control group. The bottom line shows the average of days relative to their simulated launch into space (L) which is considered as 'day 0' with the standard deviation below, or relative to the simulated return from space (R).

#	Gender	Pre-Flight	Early ISS (simulated)	Late ISS (simulated)	Early Post-Flight	Late Post-Flight
1	F	L-142	L+3	L+93	R+3	R+59
2	F	L-126	L+3	L+98	R+3	R+58
3	M	L-41	L+3	L+91	R+3	R+56
4	M	L-28	L+3	L+86	R+3	R+59
5	M	L-114	L+3	L+90	R+3	R+57
6	F	L-82	L+3	L+93	R+3	R+65
7	M	L-118	L+3	L+91	R+3	R+57
8	F	L-118	L+3	L+91	R+3	R+59
9	F	L-122	L+3	L+88	R+3	R+55
10	M	L-114	L+3	L+96	R+3	R+59
11	F	L-81	L+3	L+93	R+3	R+62
12	F	L-54	L+3	L+81	R+3	R+52
13	M	L-122	L+3	L+99	R+3	R+66
14	F	L-23	L+3	L+87	R+3	R+64
15	F	L-101	L+3	L+88	R+3	R+59
16	F	L-46	L+3	L+92	R+3	R+53
17	M	-	-	-	-	-
18	M	L-91	L+3	L+91	R+3	R+60
19	M	L-20	L+3	L+94	R+3	R+59
20	M	L-42	L+3	L+88	R+3	R+58
21	M	L-44	L+3	L+96	R+3	R+66
22	F	-	-	-	-	-
	**#M: 10<br>#F: 10**	**Ø: L-81.45d<br>±40.3d**	**Ø: L+3**	**Ø: L+91.3d<br>±4.3d**	**Ø: R+3**	**Ø: R+ 59.2d<br>±3.9d**

Supplementary Materials C – Accuracy by Distance

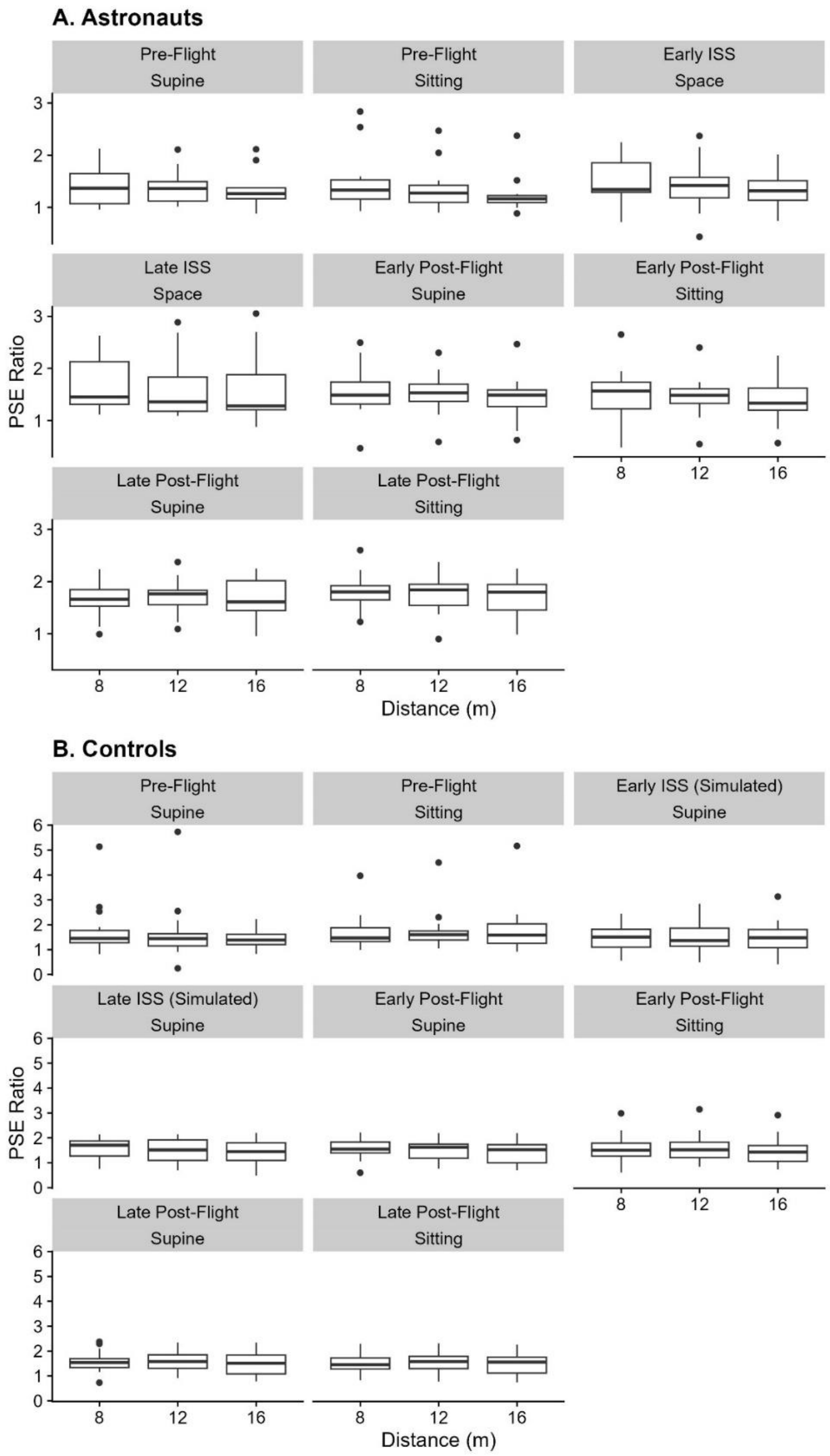

Supplementary Figure 5: Boxplots (the center line corresponds to the median; the box limits correspond to the upper and lower quartiles; the whiskers denote the 1.5x interquartile range and the points represent the outliers) of the PSEs Ratios for Astronauts (A) and Controls (B), separately per session and posture (panels) and distances (x axis).

Supplementary References

1. Shaqiri, A. *et al.* Sex-related differences in vision are heterogeneous. *Sci. Rep.* **8**, 1–10 (2018).
2. Contreras, M. J., Rubio, V. J., Pena, D., Colom, R. & Santacreu, J. Sex differences in dynamic spatial ability : *Mem Cognit* **35**, 297–303 (2007).
3. Herpers, R. *et al.* The somatogravic illusion during centrifugation: sex differences. *Front. Physiol.* **9**, 24–27 (2018).
4. Naylor, Y. & McBeath, M. Gender differences in spatial perception of body tilt. *Percept. psychophys.* **70**, 199–207 (2008).
5. Barnett-Cowan, M., Dyde, R. T., Thompson, C. & Harris, L. R. Multisensory determinants of orientation perception: Task-specific sex differences. *Eur. J. Neurosci.* **31**, 1899–1907 (2010).
6. Smith, P. F., Agrawa, Y. & Darlington, C. L. Sexual dimorphism in vestibular function and dysfunction. *J. Neurophysiol.* **121**, 2379–2391 (2019).
7. Bürkner, P. C. Advanced Bayesian multilevel modeling with the R package brms. *R J.* **10**, 395–411 (2018).
8. Andraszewicz, S. *et al.* An Introduction to Bayesian Hypothesis Testing for Management Research. *J Manage* **41**, 521–543 (2015).
